# Validation of Reference Housekeeping Genes for Gene Expression Studies in Western Corn Rootworm (*Diabrotica virgifera virgifera*)

**DOI:** 10.1371/journal.pone.0109825

**Published:** 2014-10-30

**Authors:** Thaís Barros Rodrigues, Chitvan Khajuria, Haichuan Wang, Natalie Matz, Danielle Cunha Cardoso, Fernando Hercos Valicente, Xuguo Zhou, Blair Siegfried

**Affiliations:** 1 CAPES Foundation, Ministry of Education of Brazil, Brasília, Federal District, Brazil, and Federal University of Lavras, Lavras, Minas Gerais, Brazil; 2 Department of Entomology, University of Nebraska, Lincoln, Nebraska, United States of America; 3 Agronomic Institute of Campinas, Campinas, São Paulo, Brazil; 4 Núcleo de Biologia Aplicada, Embrapa Maize and Sorghum, Sete Lagoas, Minas Gerais, Brazil; 5 Department of Entomology, University of Kentucky, Lexington, Kentucky, United States of America; Swedish University of Agricultural Sciences, Sweden

## Abstract

Quantitative Real-time PCR (qRT-PCR) is a powerful technique to investigate comparative gene expression. In general, normalization of results using a highly stable housekeeping gene (HKG) as an internal control is recommended and necessary. However, there are several reports suggesting that regulation of some HKGs is affected by different conditions. The western corn rootworm (WCR), *Diabrotica virgifera virgifera* LeConte (Coleoptera: Chrysomelidae), is a serious pest of corn in the United States and Europe. The expression profile of target genes related to insecticide exposure, resistance, and RNA interference has become an important experimental technique for study of western corn rootworms; however, lack of information on reliable HKGs under different conditions makes the interpretation of qRT-PCR results difficult. In this study, four distinct algorithms (Genorm, NormFinder, BestKeeper and delta-CT) and five candidate HKGs to genes of reference (β-actin; GAPDH, glyceraldehyde-3-phosphate dehydrogenase; β-tubulin; RPS9, ribosomal protein S9; EF1a, elongation factor-1α) were evaluated to determine the most reliable HKG under different experimental conditions including exposure to dsRNA and Bt toxins and among different tissues and developmental stages. Although all the HKGs tested exhibited relatively stable expression among the different treatments, some differences were noted. Among the five candidate reference genes evaluated, β-actin exhibited highly stable expression among different life stages. RPS9 exhibited the most similar pattern of expression among dsRNA treatments, and both experiments indicated that EF1a was the second most stable gene. EF1a was also the most stable for Bt exposure and among different tissues. These results will enable researchers to use more accurate and reliable normalization of qRT-PCR data in WCR experiments.

## Introduction

The western corn rootworm (WCR), *Diabrotica v. virgifera* LeConte (Coleoptera: Chrysomelidae), is one of the most important insect pests of cultivated maize in North America with annual losses in yield and control expenditures exceeding U.S. $1 billion annually [1], [2]. A number of strategies, such as chemical insecticides, crop rotation, biological control and transgenic plants expressing toxins from *Bacillus thuringiensis* (Bt), have been used to manage rootworm populations [3]–[6]. Bt maize has become one of the predominant management strategies [7], however, the unique capacity of rootworms to rapidly adapt to this new technology has resulted in resistance evolution and failure of the technology in some areas [8], [9]. As a result, additional transgenic technologies, such as RNA interference to knock down the expression of essential genes resulting in mortality of exposed larvae, are being developed. Expression of dsRNA in corn plants for such essential genes has caused high mortality of rootworms and results in protection of corn roots [6].

Because of interest in RNAi as a rootworm pest management tool and to identify differentially expressed genes associated with a number of different traits, including insecticide and Bt resistance, quantitative RT-PCR (qRT-PCR) has become an important research tool for WCR research. In general, with housekeeping gene(s) (HKG) as an internal reference, qRT-PCR is widely used as a standard method to evaluate the expression of target genes, including those targeted by RNAi [10], [11]. The accuracy, high sensitivity and specificity of qRT-PCR depend on many factors, such as the number of replications, primer efficiency, and the choice of appropriate reference genes [12]. The choice of appropriate reference HKGs is an essential and crucial step to allow proper interpretation of results. HKGs are constitutively expressed genes required for the maintenance of basic cellular function, and are expressed in all cells of an organism under both normal and stressful conditions [12]. Although some HKGs (such as GAPDH, HSP90, and β-actin) are expressed at relatively constant levels in most non-pathological situations [13], other HKGs may vary depending on experimental conditions [14]. Thus, finding proper reference genes is a critical and initial step in developing qPCR methods [14]. Many studies have been conducted to identify HKGs in a variety of organisms [12], [15]–[18] and some studies have suggested that there is no single reference gene that is appropriate for all variables [19]–[22].

Based on different statistical algorithms, there are four models (Genorm [12], NormFinder [23], BestKeeper [24] and delta-Ct [25]) that have been employed in reference gene evaluations. Genorm assesses the expression stability value (M) for each gene and identifies the best pair of reference genes. The program is based on the mean pairwise variation between genes across all samples and the lowest M value is considered the most stable [12]. NormFinder estimates the standard deviation for each gene and compares it with the expression of the other genes. The lowest variation between intra- and inter-group comparisons is considered the most stable gene [23]. BestKeeper is a method based on the calculation of a stability index (BKI) and provides an indication of the highest stability because it compares all genes across all samples [24]. The comparative delta-Ct method compares basic Ct values and the relative expression of ‘gene pairs’ with each sample [25].

In the work reported here, the four programs described previously were used to estimate the most efficient reference gene for *D. v. virgifera* among five commonly used genes: β-actin, GAPDH (glyceraldehyde-3-phosphate dehydrogenase), β-tubulin, *RPS9* (ribosomal protein S9), and *EF1a* (elongation factor-1α). These HKGs were tested across different larval tissues (head, midgut, fat body, integument) and development stages (eggs, first, second and third instars, female and male adults). In addition, they were tested across adults fed with dsRNAs (vATPase; Dv63a; GFP), and larvae fed with Bt toxins (Cry34; Cry 35; Cry 34/35). The results from this research provide information to define HKGs that could be used in research to evaluate gene expression in this important pest species as reference genes.

## Materials and Methods

### Biological Samples and Experiment Conditions

*D. v. virgifera* used in this study were purchased from Crop Characteristics (Farmington, MN). Adults and larvae were fed with artificial diet [26] and were reared in a growth chamber at 23±1°C and 75±5% relative humidity. The gene expression profiles were analyzed in four different experiments and included: 1) four different tissues; 2) six developmental stages; 3) two different dsRNA exposures; and 4) three different Bt toxin exposures. Five third instar larvae were dissected for four different tissues, including integument, midgut, fat body and head. The same tissues from five third instar larvae were pooled as a single replicate. All pooled tissues and whole bodies were flash-frozen in liquid nitrogen and stored at −80°C until RNA extraction. The samples for developmental stages included pooled samples of eggs, first (30 larvae), second (15 larvae) and third (6 larvae) instars, and individual female and male adults. In addition to comparisons across developmental stages and tissues, we also compared expression for two experimental conditions that potentially affect gene expression in adults and larvae; exposure to dsRNA in adults and exposure to Bt toxins in larvae. For RNAi experiments, adults were fed with artificial diet treated with double stranded RNA (dsRNA) for Gr3 (CO_2_-gustatory receptor proteins), which produces a non-lethal response and vATPase (vacuolar ATPase A), which causes mortality in rootworm adults. Water and GFP dsRNA (green fluorescent protein) were used as controls. All rootworms were exposed to treated artificial diet that provided approximately 500 ng dsRNA/beetle. This concentration has been previously shown cause gene knockdown and mortality in rootworm adults treated with vATPase dsRNA [27]. Fresh untreated diet was provided on the third day of exposure and the adults were collected on the fourth day. For the Bt toxin experiment, neonates were exposed to artificial diet treated with 15 µg/cm^2^ of either Cry34Ab1, Cry35Ab1, or with 15 µg/cm^2^ of both Cry34Ab1 and Cry35Ab1 combined for 24 hrs. Control treatments consisted of diet treated with 20 µM sodium acetate, pH 3.5 which was used to dilute the different toxin preparations. Although individual toxins have limited activity by themselves [28], the combination of Cry34/45Ab1 at 15 µg/cm^2^ for both toxins causes significant growth inhibition (personal observation). Each treatment condition was repeated with three different preparations.

### RNA Extraction, Reverse Transcription and Primer Design

Total RNA was extracted using the RNeasy Mini Kit (Qiagen) according to the manufacturer's instructions. The RNA integrity was confirmed by agarose gel electrophoresis and measurement of the absorbance ratio of 260/280 nm using a Nanodrop spectrophotometer (Thermo Scientific, Franklin, MA). QuantiTect Reverse Transcription kit (QIAGEN) was used for cDNA synthesis with 1000 ng/µL of RNA. Gene specific primers for each housekeeping gene were designed using Primer3Plus (http://www.bioinformatics.nl/cgi-bin/primer3plus/primer3plus.cgi/). Descriptions of each primer are provided in Table 1. PCR amplification efficiencies (E) and correlation coefficients (R^2^) were checked to validate the primers. Standard curves were constructed using 5-fold serially diluted cDNA for each primer pair.

**Table 1:** General information of the five candidate HKGs.

Gene name	Primer Sequence (5′-3′)	*D. v. virgifera* homolog locus	Function	Amplicon (bp)	E (%)	R^2^
**TUB - β-tubulin**	Forward: TTGAGTTGCCGATGAAAGTG	XM_962174.1	Involved in microtube structures	205	97.9	0.999
	Reverse: GATCCCAGACACGGAAGGTA					
**EF1a - elongation factor 1α**	Forward: ACCAGATTTGATGGCTTTGG	XM_003705302.1	Bringing of aminoacyl-transfer RNA to the ribosome	194	97.7	0.999
	Reverse: CACCCAGAGGAGCTTCAGAC					
**GAPDH - glyceraldehydes-3 phosphate dehydrogenase**	Forward: TTGTGGTGAACACTCCGGTA	XM_004258320.1	Carbohydrate metabolism	154	95.6	0.998
	Reverse: GGTCGCTACAAGGGATGTGT					
**Actin - β-actin**	Forward: TCCAGGCTGTACTCTCCTTG	NM_001164951.1	Involved in cell mortality, structure and integrity	133	94	0.997
	Reverse: CAAGTCCAAACGAAGGATTG					
**RPS9 - 40S ribosomal protein S9**	Forward: AATGTGTCGTTGTCTGAT	XM_001608220.2	Component of the 40S subunit of ribosome	170	100.7	0.988
	Reverse: GTCGTTTGGTTCGTATTG					

R^2^: Correlation Coefficients; E: Amplification efficiency.

### Real-Time PCR

The PCR mixture contained 1 µL of a 1∶1 dilution of the synthesized cDNA (1∶10 dilution for different tissues experiment), 0.2 µL of each primer (diluted to 10 µM), 5 µL of the SYBR Green PCR Master Mix (Applied Biosystems, USA) and 3.6 µL of ddH_2_O. All reactions were carried out in duplicate for each cDNA template with a final volume of 10 µL. qRT-PCR assays were performed using the 7500 Fast Real-Time PCR system (Applied Biosystems) and SYBR Green (Applied Biosystems). The PCR amplifications were conducted with the following cycling conditions: one cycle at 95°C (20 s), followed by 40 cycles of denaturation at 95°C (3 s), annealing and extension at 60°C for 30 s. At the end of each PCR reaction, a melting curve was generated to confirm a single peak and rule out the possibility of primer-dimers and non-specific product formation.

### Expression Stability Analysis of Candidate Reference Genes

A web based tool (RefFinder (www.leonxie.com/referencegene.php) which integrated all four software algorithms, GeNorm [12], NormFinder [23], BestKeeper [24] and the comparative delta-Ct method [25] was used to evaluate reference gene stability from the experimental datasets [29]. The mean Ct value of each sample and for each primer was calculated by equation 2^(−ΔΔCt)^ and was used as input data on the website [30].

## Results

### Primer Specificity and Efficiency

All PCR amplifications of each primer were confirmed by the presence of a single peak in melting curve analyses and a specific band with expected size based on agarose electrophoresis (data not shown). A primer efficiency value between 93.9% and 100.6% was displayed for all primers with a correlation coefficient (R^2^) ranging around 0.99 and their specificity was verified with the BLAST program (Table 1).

### Cycle Threshold (Ct) Values of Reference Housekeeping Genes

**Figure 1:**
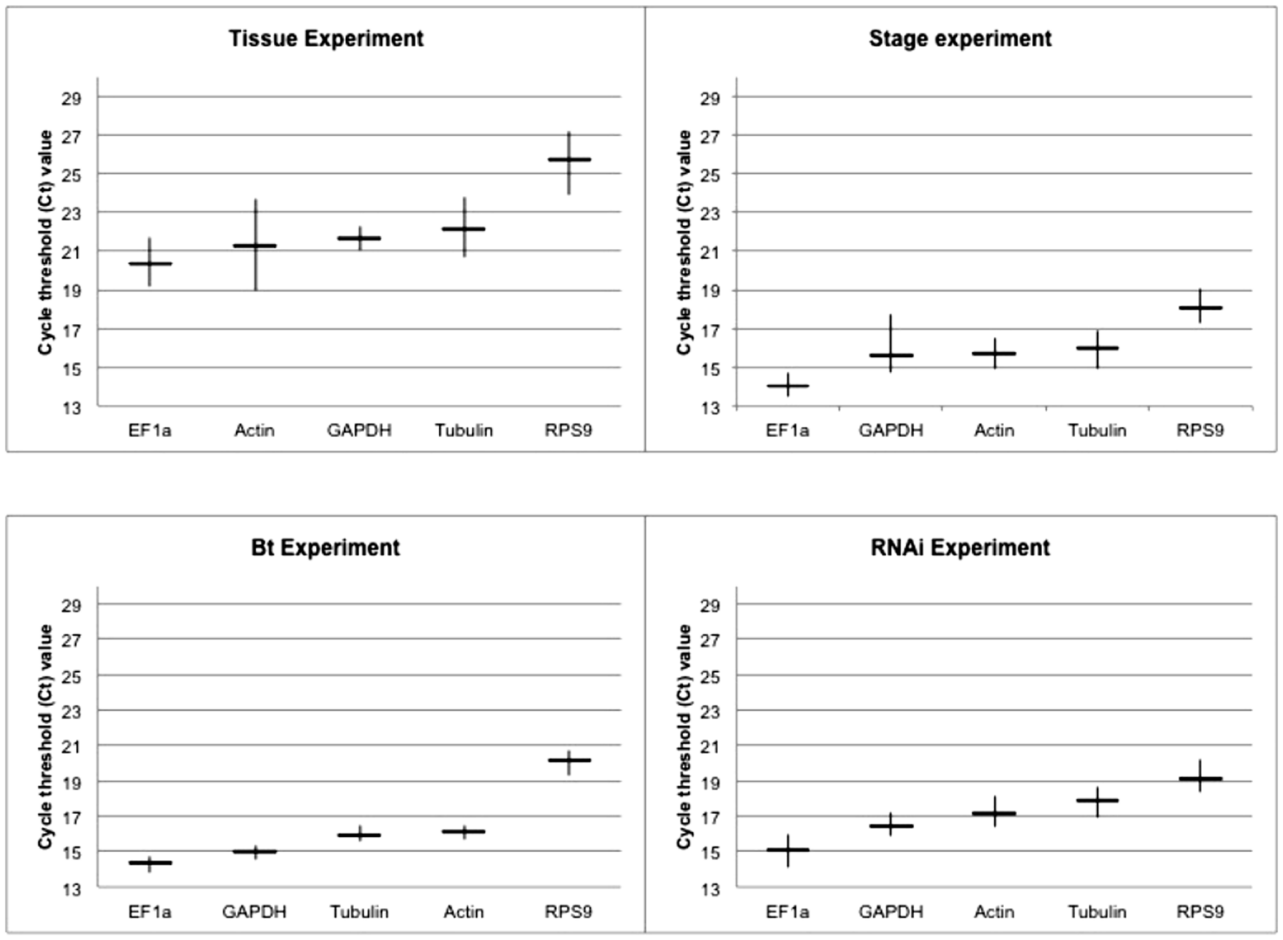
Ct values of the five candidate reference genes in all four experiments. Black bars indicate an average between maximum (Max) and minimum (Min) Ct values.

The expression profiles of all qRT-PCR products for all primers and all experiments are shown in Figure 1. The expression levels of all HKGs were measured by the Ct value, which is the number of PCR cycles needed to reach a specific threshold level of detection and is inversely correlated with the quantity of initial RNA template. For qRT-PCR normalization, a moderately expressed reference gene is preferred because extremely high or low expression of a HKG could introduce variability to data analysis [31], so a standard Ct value range was analyzed for all four experiments (Fig 1). EF1a was the most highly expressed HKG and RPS9 the least expressed HKG. GAPDH, tubulin and actin were moderately expressed when compared among other HKGs.

The WCR tissues included in the experiment were integument, midgut, fat body and head. The five analyzed HKGs exhibited a range of expression between 18 and 27 cycles among the different tissues analyzed. EF1a showed average Ct values below 21 cycles, and actin, GAPDH and tubulin were below 23 cycles. RPS9 was the least expressed HKG, with Ct value between 24 and 27 cycles.

Among the development stages analyzed, the five HKGs showed a range of expression between 13 and 19 cycles. EF1a was the most highly expressed HKG, with average Ct values below 15 cycles. GAPDH, tubulin, and actin showed average Ct value below 17 cycles and RPS9 was the least expressed HKG, with Ct value between 17 and 19 cycles.

The HKGs from adults fed with artificial diet treated with dsRNA for Gr3 (CO_2_-gustatory receptor proteins), vATPase (vacuolar ATPase A) and controls showed a range of expression between 14 and 20 cycles. EF1a was the most highly expressed HKG, showing an average expression below 15 cycles and RPS9 was the least expressed HKG, with Ct values between 19 and 21 cycles. GAPDH, tubulin and actin showed an average Ct value below 17 cycles.

The five analyzed HKGs from neonate larvae exposed to Bt toxins and the buffer control showed a range of expression between 13 and 20 cycles. EF1a and GAPDH showed an average expression below 17 cycles. Actin and tubulin showed average Ct value below 19 cycles and RPS9 showed the Ct values in the range of 18 and 20 cycles.

### Stability Analysis of Candidate Reference Genes

Four different programs were used for analysis of reference gene expression (geNorm, NormFinder, BestKeeper, and delta-Ct method) to estimate the stability of five candidate reference housekeeping genes under four different experimental conditions using a web tool that provides a reference gene stability ranking. The final ranking is based on the calculation of the geometric mean of the four algorithms; the smaller the geometric mean, the greater the stability of reference gene expression [32].

The first experiment compared gene expression among different tissues. The geNorm program was used to calculate the stability of the reference genes based on an ‘M’ value. The lower the *M* value, the more stable is the expression of the reference gene, and values of *M* that surpass the cutoff value of 1.5 are not considered stable across treatments. According to this algorithm, EF1a and GAPDH were the most stable genes with an *M* score of 0.522, and actin was the only gene slightly above the cutoff with an *M* score of 1.536. The NormFinder program analyzes both intra and inter-group variations and lower output scores indicate reduced variation of the reference gene expression. For different tissues, NormFinder identified EF1a as a most stable reference gene with a stability value of 0.261. The BestKeeper algorithm calculates standard deviation (SD), with lower values considered more stable, and values that surpass the cutoff value (*SD* <1) are considered to be unstable across all treatments. This analysis indicated both actin and RPS9 exceeded the cutoff value, while GAPDH was the most stably expressed reference gene with a SD value of 0.46. The comparative delta-Ct method was used to estimate the most stably expressed reference gene based on delta-Ct value variation. A higher value is considered more stable and the results were similar to NormFinder, with the EF1a value of 1.133. The final ranking suggests that the most stable reference gene was EF1a followed by GAPDH, tubulin, RPS9, and actin (Table 2) although there were only a few instances where the calculated stability values exceeded recommended values.

**Table 2:** Ranking of the candidate HKGs according to their stability value by geNorm, NormFinder and BestKeeper analysis in Tissue Experiment.

Gene name	Stability value “M” (geNorm)	Ranking order (geNorm)	Stability value (NormFinder)	Ranking order (NormFinder)	Stability value “SD” (BestKeeper)	Ranking order (BestKeeper)	Stability value (delta-CT)	Ranking order (delta-CT)	Stability value (comprehensive)	Ranking order
**TUB**	0.681	3	0.626	3	0.84	3	1.269	2	2.71	3
**EF1a**	0.522	1	0.261	1	0.60	2	1.133	1	1.19	1
**GAPDH**	0.522	1	0.859	4	0.46	1	1.325	4	2.00	2
**Actin**	1.536	5	2.621	5	1.90	5	2.677	5	5.00	5
**RPS9**	0.774	4	0.371	2	1.06	4	1.273	3	3.13	4

For different life stages, the geNorm statistic algorithm indicated that EF1a and actin were the most stable genes with an *M* score of 0.485. Actin with a value of 0.268 and SD value of 0.432 was also identified as the most stable reference gene by NormFinder and BestKeeper algorithms, respectively. The comparative delta-Ct method indicated that EF1a was the most stable gene, with a value of 0.669. The geometric mean ranking showed actin as the most stable gene, followed by EF1a, RPS9, tubulin, and GAPDH as the least stable gene (Table 3).

**Table 3:** Ranking of the candidate HKGs according to their stability value by geNorm, NormFinder and BestKeeper analysis in Stage Experiment.

Gene name	Stability value “M” (geNorm)	Ranking order (geNorm)	Stability value (NormFinder)	Ranking order (NormFinder)	Stability value “SD” (BestKeeper)	Ranking order (BestKeeper)	Stability value (delta-CT)	Ranking order (delta-CT)	Stability value (comprehensive)	Ranking order
**TUB**	0.603	4	0.719	4	0.556	4	0.867	4	4.00	4
**EF1a**	0.485	1	0.291	2	0.494	2	0.669	1	1.414	2
**GAPDH**	0.789	5	0.987	5	0.593	5	1,069	5	5.00	5
**Actin**	0.485	1	0.268	1	0.432	1	0.672	3	1.316	1
**RPS9**	0.485	3	0.291	3	0.556	3	0.669	2	2.711	3

Table 4 summarizes data obtained from the Bt experiment. The geNorm statistic algorithm showed that tubulin and GAPDH were the most stable genes with an *M* score of zero. EF1a was identified as the most stable reference gene with a stability value of 0.18 and SD value of 0.403 using NormFinder and BestKeeper algorithms respectively. The comparative delta-Ct method indicated that GAPDH was the most stable gene, with a stability value of 2.913. The final ranking calculated based on the combined algorithm values from the most to the least stable genes was EF1a, GAPDH, tubulin, actin, and RPS9, although all genes exhibited relatively stable expression.

**Table 4:** Ranking of the candidate HKGs according to their stability value by geNorm, NormFinder and BestKeeper analysis in Bt Experiment.

Gene name	Stability value “M” (geNorm)	Ranking order (geNorm)	Stability value (NormFinder)	Ranking order (NormFinder)	Stability value “SD” (BestKeeper)	Ranking order (BestKeeper)	Stability value (delta-CT)	Ranking order (delta-CT)	Stability value (comprehensive)	Ranking order
**TUB**	0	1	0.42	3	0.458	4	2.115	2	2.213	3
**EF1a**	0.383	4	0.18	1	0.403	1	2.213	3	1.861	1
**GAPDH**	0	1	0.5	4	0.558	5	1.861	1	2.115	2
**Actin**	0.261	3	0.32	2	0.443	3	2.913	4	2.913	4
**RPS9**	0.453	5	0.5	5	0.403	2	3.976	5	3.976	5

The geNorm algorithm showed for the RNAi experiment that EF1a and RPS9 were the most stable genes with an *M* score of 0.289. NormFinder identified RPS9 as a most stable reference gene with a stability value of 0.107. The BestKeeper algorithm indicated that GAPDH was the most stably expressed reference gene with a SD value of 0.167. The comparative delta-Ct method showed similar results to NormFinder, with RPS9 exhibiting the most stable expression (stability score value of 1.316). From the most to the least stable reference genes, the geometric mean ranking was RPS9, EF1a, GAPDH, actin, and tubulin (Table 5).

**Table 5:** Ranking of the candidate HKGs according to their stability value by geNorm, NormFinder and BestKeeper analysis in RNAi experiment.

Gene name	Stability value “M” (geNorm)	Ranking order (geNorm)	Stability value (NormFinder)	Ranking order (NormFinder)	Stability value “SD” (BestKeeper)	Ranking order (BestKeeper)	Stability value (delta-CT)	Ranking order (delta-CT)	Stability value (comprehensive)	Ranking order
TUB	0.468	5	0.483	5	0.681	5	0.559	5	5.00	5
EF1a	0.289	1	0.21	2	0.444	2	0.415	2	1.682	2
GAPDH	0.407	4	0.431	4	0.167	1	0.526	4	2.828	3
Actin	0.335	3	0.324	3	0.556	4	0.457	3	3.224	4
RPS9	0.289	1	0.107	1	0.5	3	0.38	1	1.316	1

## Discussion

One of the most important technologies used to quantify gene expressions involves qRT-PCR [10]. In order for qRT-PCR experiments to provide reliable estimates of gene expression, reference genes should exhibit stable expression throughout the life of the target organism and among different experimental conditions [12]. Therefore, identifying appropriate reference housekeeping genes is critical and one of the main considerations in designing an experiment [14] that compare gene expression. Many genes have been considered as reference genes across different treatments, various tissues, and developmental stages [13]. However, some qRT-PCR normalization studies have reported a lack of stable expression of those genes among variables [12], [15] and a number of studies have suggested that there is no single reference gene for all these variables [19]–[22].

To our knowledge, this is the first study to validate a set of candidate reference genes for qRT-PCR in *D. v. virgifera* by several algorithms (geNorm, NormFinder, BestKeeper, delta-Ct methods, and geometric mean ranking) under different experimental conditions (different tissues, stages, feeding adults with dsRNA and feeding larvae with Bt toxins). Our results suggest that EF1a is the most stable reference gene for Bt toxin exposures, different tissues and the second most stable gene for RNAi experiments and different developmental stages. Although the EF1a gene has rarely been used as a reference gene, some recent studies have indicated its suitability as a reference housekeeping gene in insects [16], [33]–[35]. The actin gene has been widely used as a reference gene among a number of different insect species and experiments [35]–[39], although recent studies have suggested that the stability of actin may make it unsuitable as a reference housekeeping gene for certain comparisons [21], [40]–[41]. Our studies would suggest that β-actin may not be suitable to compare expression among different tissues but is appropriate for other comparisons.

When we consider the outcomes of the four analyses, geNorm and NormFinder produced similar results in almost all experiments. However, our analyses did not produce a common result for all algorithms and for different conditions. Although the results were similar among some treatments and analyses, the relation between the most stable reference gene and different experiments were unique and specific. Similar studies of reference genes for qRT-PCR from different insect species and conditions are consistent with our results [16], [20], [21]. In conclusion, we tested five reference gene candidates in four different experiments and with four statistical algorithms. The results generated were used to produce a final ranking of all experimental systems. The EF1a gene was considered the most stable reference gene for both Bt exposures and different tissues. β-actin was considered the best reference gene in different experiments involving different life stages. For RNAi experiments, the RPS9 was considered the reference gene with highest stability in expression. These results suggest that there is no single reference gene suitable for all comparisons and for *D. v. virgifera*, reference genes can respond differently to different experiments.
