# Correction: Validation of Reference Housekeeping Genes for Gene Expression Studies in Western Corn Rootworm (*Diabrotica virgifera virgifera*)

**DOI:** 10.1371/journal.pone.0124187

**Published:** 2015-04-07

**Authors:** The PLOS ONE Staff

The first author’s name is spelled incorrectly in the citation. The correct citation is: Rodrigues TB, Khajuria C, Wang H, Matz N, Cunha Cardoso D, Valicente FH, et al. (2014) Validation of Reference Housekeeping Genes for Gene Expression Studies in Western Corn Rootworm (*Diabrotica virgifera virgifera*). PLoS ONE 9(10): e109825. doi:10.1371/journal.pone.0109825
